# DNA hypermethylation of Fgf16 and Tbx22 associated with cleft palate during palatal fusion

**DOI:** 10.1590/1678-7757-2018-0649

**Published:** 2019-10-07

**Authors:** Xuan Shu, Zejun Dong, Liuhanghang Cheng, Shenyou Shu

**Affiliations:** 1 Second Affiliated Hospital of Shantou University Medical College, Cleft Lip and Palate Treatment Center, Shantou, Guangdong, China.

**Keywords:** DNA methylation, Cleft palate, Gene expression

## Abstract

**Objective:**

Cleft palate (CP) is a congenital birth defect caused by the failure of palatal fusion. Little is known about the potential role of DNA methylation in the pathogenesis of CP. This study aimed to explore the potential role of DNA methylation in the mechanism of CP.

**Methodology:**

We established an all-*trans* retinoic acid (ATRA)-induced CP model in C57BL/6J mice and used methylation-dependent restriction enzymes (MethylRAD, FspEI) combined with high-throughput sequencing (HiSeq X Ten) to compare genome-wide DNA methylation profiles of embryonic mouse palatal tissues, between embryos from ATRA-treated *vs*. untreated mice, at embryonic gestation day 14.5 (E14.5) (n=3 *per* group). To confirm differentially methylated levels of susceptible genes, real-time quantitative PCR (qPCR) was used to correlate expression of differentially methylated genes related to CP.

**Results:**

We identified 196 differentially methylated genes, including 17,298 differentially methylated CCGG sites between ATRA-treated *vs*. untreated embryonic mouse palatal tissues (P<0.05, log_2_FC>1). The CP-related genes Fgf16 (P=0.008, log_2_FC=1.13) and Tbx22 (P=0.011, log_2_FC=1.64,) were hypermethylated. Analysis of Fgf16 and Tbx22, using Gene Ontology (GO) and the Kyoto Encyclopedia of Genes and Genomes (KEGG), identified 3 GO terms and 1 KEGG pathway functionally related to palatal fusion. The qPCR showed that changes in expression level negatively correlated with methylation levels.

**Conclusions:**

Taken together, these results suggest that hypermethylation of Fgf16 and Tbx22 is associated with decreased gene expression, which might be responsible for developmental failure of palatal fusion, eventually resulting in the formation of CP.

## Introduction

Cleft palate (CP) is a congenital birth defect caused by both environmental and genetic factors.^1^ It is universally acknowledged that palatal fusion is the most crucial process in the palate formation. In a mouse, palatal shelves appose at the midline and palatal fusion occurs at E 14.5, and an imbalance of embryonic palatal mesenchyme cell proliferation and apoptosis might result in CP.^2^ Previous studies have demonstrated that Fgf16 and Tbx22 participate in murine palate development.^3^ However, the role of Fgf16 and Tbx22 during palatal fusion is still unknown.

DNA methylation is an important epigenetic modification and plays a crucial role in many biological processes, such as embryogenesis, cellular differentiation, X-chromosome inactivation, genomic imprinting and transcriptional silencing.^4^ Methylation patterns of specific genes have been found to undergo dynamic changes in embryonic development and contribute to tissue-specific gene expression.^5^ The DNA methylation pattern of a mouse undergoes dynamic and widespread alterations during palatogenesis, and failing to establish correct methylation patterns can result in CP,^6^ which suggests that CP-susceptible genes (e.g. Fgf16 and Tbx22) in embryonic mice provide new clues to epigenetic markers involved in CP. Nevertheless, details on the methylation patterns of CP-susceptible genes during palatal fusion are very limited, and the methylation pattern of Fgf16 and Tbx22 underlying palate development and its contribution to CP is still unclear.

To explore the potential involvement of DNA methylation in regulating palatal fusion, we previously established a CP model in which pregnant C57BL/6J mice are treated with all-*trans* retinoic acid (ATRA) to introduce CP in the embryos.^7^ ATRA is a vitamin A metabolite and functions to support normal pattern formation during embryogenesis. Abnormally high concentrations of ATRA may affect palatogenesis by interfering in medial edge epithelia (MEE) cell differentiation and apoptosis, including inhibition of mesenchymal proliferation and signaling growth factors (transforming growth factor-β and platelet-derived growth factor).^8^ Cuervo, et al.^9^ (2002) reported that high ATRA concentration blocked palatal shelf fusion and increased apoptosis within the MEE cell adhesion process, and which may result in fetal malformations, including cleft palate, in both experimental animals and humans. We integrated DNA methylation analysis of Fgf16 and Tbx22, by MethylRAD-seq, with their biological characteristics identified by GO and KEGG pathway enrichment analysis. The restriction enzyme FspEI recognizes 5-methylcytosine and 5-hydroxymethylcytosine in CCGG and CCWGG (where W=A or T),^10^ and cleaves DNA bilaterally to generate 32 bp fragments at a methylated CCGG site and 31 bp fragments at a methylated CCWGG site, with either FspE1 recognition site in the middle. The qPCR was used to correlate Fgf16 and Tbx22 expression levels with methylation levels to elucidate the molecular regulatory mechanisms underlying the development of CP.

## Methodology

### Ethics, animals and treatment

This study was approved by the Laboratory Animal Ethical Committee of the Medical College of Shantou University (SUMC2015-106; Guangdong, China). C57BL/6J mice of 20–28 g in body weight and 8 to 10 weeks of age were purchased from the Beijing Vital River Laboratory Animal Technology Co. Ltd. (Beijing, China). In this study, female mice were mated with male mice of similar weight and age overnight. Embryonic gestation day 0.5 (E0.5) was designated to be at 8 AM the next day when a vaginal plug was observed. Pregnant mice at E10.5 were randomly divided into an all-*trans* retinoic acid (ATRA, Sigma-Aldrich, St. Louis, MO, USA)-treated and control (mock-treated) group. Mice in the ATRA-treated group were treated, via oral gavage, with ATRA at 70 mg/kg dissolved in corn oil as described previously.^7^ The untreated group was given an equivalent volume of corn oil. At E14.5, mice were euthanized, and the embryonic palatal shelves (3 ATRA-treated samples *vs.* 3 untreated samples) were resected and stored at −80°C until use.

### DNA preparation, library construction and MethylRAD-seq

Genomic DNA was extracted from palatal shelf tissues using the conventional cetyltrimethyl ammonium bromide (CTAB) method following the manufacturer's instructions (AMRESCO Inc, Solon, OH, USA), and MethylRAD library construction and sequencing were the same as previously reported.^10^ Paired-end sequencing was performed on a HiSeq X Ten platform (100-150 bp) (Illumina Inc, San Diego, CA, USA), according to the manufacturer's protocol, by Shanghai Oebiotech Co. Ltd. (Shanghai, China).

### MethylRAD-seq analysis

After quality-control and filtering of the original reads, high-quality reads were mapped against these reference sites (ftp://ftp.ensembl.org/pub/release-84/fasta/mus_musculus/dna/Mus_musculus.GRCm38.dna.toplevel.fa.gz) using the SOAP program.^11^ In order to improve accuracy in the follow-up analysis, Pear software (v0.9.6)^12^ was used to re-filter paired-end sequencing by removing: (i) low quality reads (Phred quality score lower than 30) and (ii) sequences containing too many N bases (sites containing more than 8% of N bases). Signatures containing FspEI sites were extracted from the genome as the reference sequence. Sites covered by at least three reads were regarded as authentic methylated sites. The number of methylated sites and the depth of signature coverage of each sample were then calculated. The untranslated region (UTR) was calculated using snpEff software (version: 4.3p),^13^ and was counted using bed tools software (v2.25.0)^14^ according to the annotation document and the distribution of methylation sites in the different gene elements in each sample. Relative quantification of DNA methylation levels for sites and genes was determined using the normalized read depth (reads *per* million, RPM). The methylation level between genes was assessed based on the sequencing depth information and each site of relative quantitative methylation level, using R package edge R.^15^ For assessing the methylation level of genes between ATRA-treated *vs*. untreated for the three biological replicates, we implemented cluster analysis to further identify the changes in gene methylation level between the groups.

### GO and KEGG enrichment analysis

GO and KEGG analysis were performed using the MethylRAD data.^15^,^16^ GO analysis was used to visualize the biological process and molecular function. KEGG analysis identified cell signaling pathways. The number of genes included in each GO and KEGG category was calculated and the statistical significance of gene enrichment was counted using the hypergeometric distribution test and p-values were adjusted for multiple testing correction.

### Protein-protein interaction (PPI) network construction

In this study, we used the online database, The Search Tool for the Retrieval of Interacting Genes (STRING) (https://string-db.org/cgi/input.pl), to construct the PPI network of differentially methylated genes. Then the PPI network was constructed and visualized using Cytoscape 3.5.1.

### Validation of susceptibility gene expression by qPCR

The same samples used for MethylRAD-seq were used for reverse transcription. Briefly, total RNA was isolated from mouse palatal shelf tissues and reverse-transcribed into cDNA using TRIzol reagent (Invitrogen Inc, Carlsbad, CA USA) and the Thermo First cDNA Synthesis Kit (Thermo Scientific^™^, Waltham, MA, USA), respectively, according to the manufacturers' protocols. In each qPCR tube, a 20 μl reaction mix was prepared using 2×SG Green qPCR Mix (with ROX) (Sino Gene, Beijing, China). The thermal profile was 40 cycles of 95°C for 10 seconds and 60°C for 30 seconds, followed by a dissociation curve check. The qPCR primer sequences for Fgf16 were as follows: forward, 5′ACGTGAATGTGTTTTCCGGG3′, reverse, 5′CCGTCTTTATTCAGGGCCAC3′. The qPCR primer sequences for Tbx22 were as follows: forward, 5′GACCTACCCATGGATGCCTT3′, reverse, 5′GTCACTGGAGATGAGCCACT3′. The 2-^ΔΔ^Ct method was used to calculate mRNA expression changes,^17^ and β-actin was used as an internal control. All reactions were carried out in triplicate for technical and biological repetitions. Statistical analysis was performed via *t*-tests using SPSS 16.0 statistical software.

### Statistical analysis

The correlations of methylation levels between samples were evaluated by calculating the Pearson's correlation coefficients. Methylation level between groups and genes was assessed using R package edge R.^15^ All statistical analysis of PCR-data was performed using Student's *t*-test to compare the means between two groups. The differential p-value (P<0.05) and fold change (log_2_FC>1) between different sites and genes were considered functionally relevant.

## Results

### Morphology and histology of embryonic palate shelf tissue

In palate shelf tissue and histological sections of untreated E14.5 embryos, it can be observed that the palatal shelf has already contacted the midline and has fused to form the midline epithelial seam (MES) in the mid-anterior region, whereas in palate shelf tissue and histological sections from ATRA-treated embryos, the palatal shelf remained completely separated without fusion.

### DNA methylation in E14.5 embryonic palatal shelves from ATRA-treated *vs*. untreated

**Figure 1:**
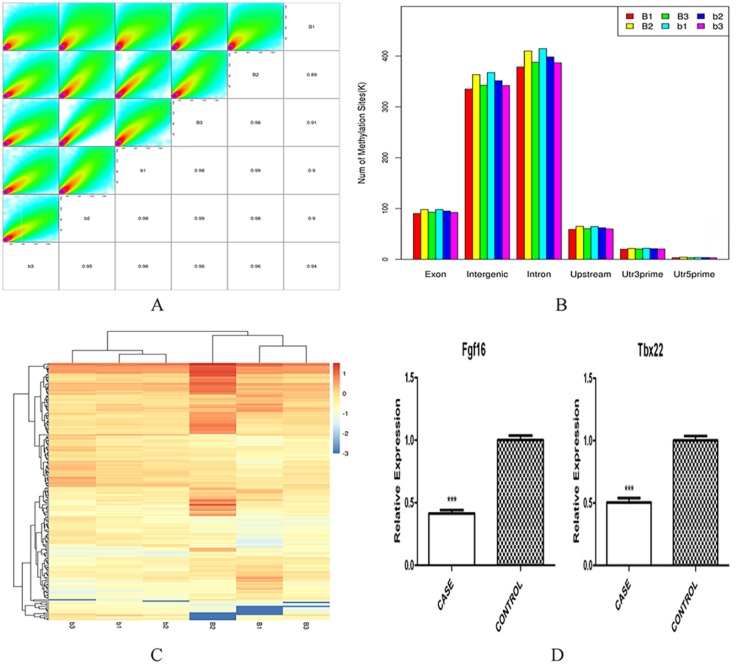
A: Scatter plots of the methylation level between samples. The left upper triangular region is the scatter plot of the methylation level of the two samples. The lower right triangular region corresponds to the Pearson's correlation coefficient, and the diagonal line is the sample name (R value: 0.89-0.99). B: Distribution in different components of the genome. The Y-axis shows the number of methylation sites. The X-axis shows the different components of the genome. C: Hierarchical cluster analysis heat-map of differentially-methylated genes between ATRA-treated *vs*. untreated. D: Relative expression levels of Fgf16 and Tbx22 at E14.5, between ATRA-treated *vs*. untreated, using qPCR and normalized to β-actin. Bars marked with different letters are significantly different from each other, data show mean ± SEM (***P<0.001)

The results for the Pearson's correlation analysis are presented in Figure 1A. The methylation level between groups was strongly correlated (*R* value: 0.89-0.99). In this study, we generated a total of 196 differentially methylated genes, including 118 hypermethylated genes and 78 hypomethylated genes between ATRA-treated *vs*. control embryonic mouse palatal tissues. Among the differentially methylated genes, 17,298 differentially methylated CCGG sites were identified, and most of the sites were concentrated in intergenic and intron regions, with a relatively small portion of methylation sites being allocated to other functional components of the genome (Figure 1B). Hierarchical cluster analysis on differentially methylated genes showed the methylation level of genes among ATRA-treated was higher than that in the control. Hypomethylated genes were clustered near the bottom, whereas hypermethylated genes were clustered near the top (Figure 1C).

### GO and KEGG enrichment analysis

**Figure 2:**
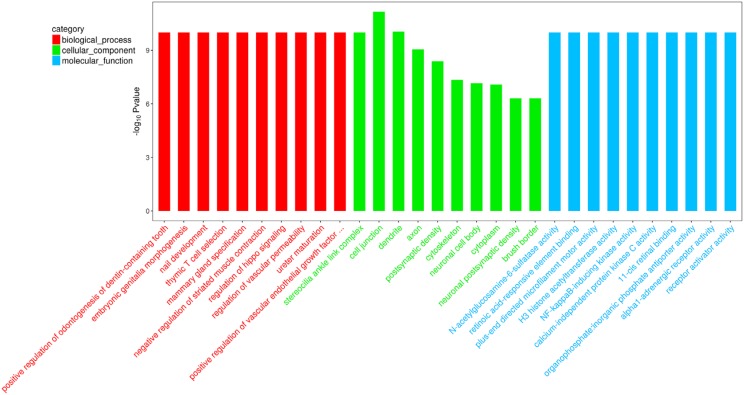
GO enrichment analysis of differentially-methylated genes, including cellular component, molecular function and biological process

**Figure 3:**
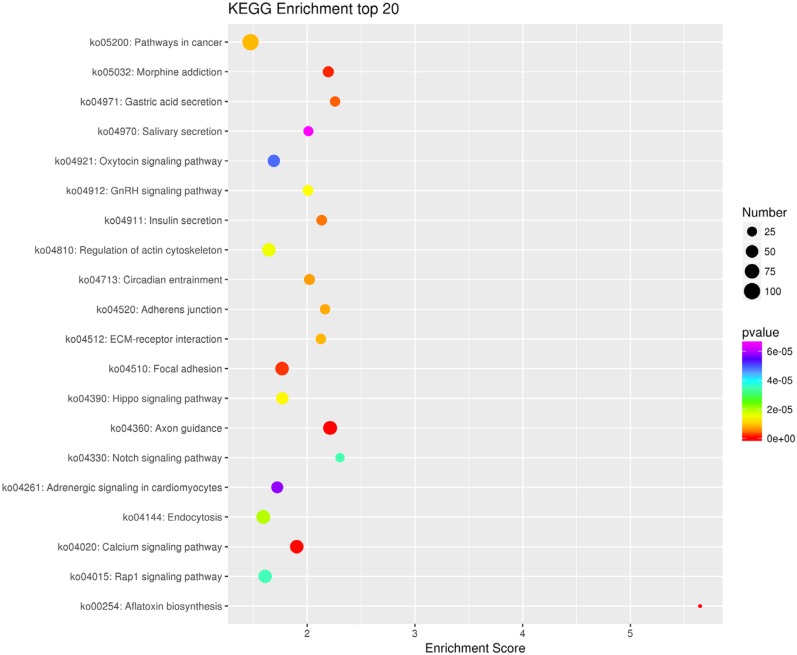
KEGG enrichment of the top 20 bubble diagrams of differential methylation-related genes. The X-axis is the enrichment score, the bigger the bubble, the more genes they contain. The color of bubbles varied from red-blue-green to yellow, and the concentration indicated by p-value is as large as the enrichment score. The X-axis comprises the cell signal pathways

GO and KEGG analysis were performed to classify the functions and the most prominent pathways of the 196 differentially methylated genes. The results of the GO analysis (Top 30) were shown in Figure 2 and further identified that the following GO analysis were significantly associated with palatal fusion, including biological process for the “regulation of hippo signaling,” and “positive regulation of vascular endothelial growth factor signaling pathway,” cellular component for the “cell junction,” and “cytoskeleton,” molecular function for the “H3 histone acetyltransferase activity.” The results of the KEGG pathway analysis (Top 20) were shown in Figure 3 and further KEGG pathway analysis identification indicated that the following pathways were significantly associated with palatal fusion, including “Calcium signaling pathway”, “Focal adhesion”, “Adherens junction”, “Hippo signaling pathway”, and “Notch signaling pathway”. To further study the correlation between DNA methylation of susceptibility genes and CP during palatal fusion, using the MethylRAD-seq data, we screened for genes that fulfilled the following conditions: i) CP-related genes show differential methylation at site that must be met; ii) the trend of change of methylation at methylated sites must correspond to the gene methylation level; iii) enrichment for a CP-related signaling pathway. Only Fgf16 and Tbx22 met the requirements above. We further analyzed and identified GO and KEGG analysis for Fgf16 and Tbx22. For Fgf16, GO analysis for the biological process category showed enrichment for “fibroblast growth factor receptor signaling pathway,” and molecular function showed enrichment of the terms “fibroblast growth factor receptor binding.” KEGG pathway analysis for Fgf16 showed the “MAPK signaling pathway”. GO in the molecular function category for Tbx22 only showed enrichment for “transcription factor activity, sequence-specific DNA binding” (P<0.05).

### Identifying differentially methylated levels from site to gene in ATRA-treated *vs*. untreated

We thoroughly examined the list of significantly differentially methylated sites located within genes that are related to the development of the embryonic palate. Fgf16 and Tbx22 were among the sequences of differentially methylated sites screened based on annotation and method of sequence alignment. The positions of differentially methylated sites in Fgf16 were located within CCGG sequences of intergenic regions (Chromosome X: 105725515- 105764278/105774940-105797614) and included the promoter (Chromosome X: 105763200-105765801) and CCCTC binding factor (CTCF) (Chromosome X: 105762801-105763200/105,786,401- 105,786,800) regions. For Tbx22, methylation was within CCGG exon sequences and the 3′ and 5′ UTR, with all sites being hyper-methylated in the ATRA-treated embryos.

### PPI network analysis

The STRING database was used to construct a PPI network for the differentially methylated genes, including 118 hypermethylated genes and 78 hypomethylated genes. Fgf16 and Tbx22 were located at the edge of PPI network. Fgf16 associated with two hub genes Cetn2 and Uprt.

### The qPCR validation of methylated Fgf16 and Tbx22

According to the MethylRAD-seq results showing increased methylation in the Fgf16 and Tbx22 genes, qPCR for Fgf16 and Tbx22 were performed to determine their mRNA expression levels. The results indicated that expression for both Fgf16 and Tbx22 was lower in CP embryonic mouse palatal tissues than in untreated embryonic mouse palatal tissues (P<0.001, Figure 1D). When comparing mRNA expression with the methylation results, reciprocal relationships were found in the embryonic palates from ATRA-treated mice, indicating increased Fgf16 and Tbx2 methylation levels, but decreased gene expression when compared with untreated, i.e. the change in expression level negatively correlated with methylation level during palatal fusion.

## Discussion

DNA methylation is one of the most common epigenetic events and participates in establishing and maintaining chromatin structure, and regulates gene transcription during palatal fusion.^18^ There are three major aspects of the molecular control of palatal fusion, i.e. global genomic alterations, methylation from site to gene-level alterations, and their impact on gene expression.^19^ Seelan, et al.^20^ (2012) discussed the differentially methylated regions (DMRs) and Seelan, et al.^21^ (2013) used Nimble Gen 2.1M mouse promoter arrays to perform a methylome analysis CP-related DMRs. Kuriyama, et al.^22^ (2008) also discussed the status of DNA methylation within CpG islands and in global DNA by cytosine extension assay and restriction landmark genomic scanning. Liu X, et al.^23^ (2016) only discussed the CpG-9 site in the TGF-β3 promoter region-1. Xuan Shu, et al.^24^ (2018) only discussed a non-CpG site within the HDAC4 gene. In this study, we performed a genome-wide DNA methylation analysis in embryonic mouse palatal shelf tissues and further identified differentially methylated CP-related genes from the site-level to the gene-level by comparing DNA methylation in E14.5 embryonic palate tissue from ATRA-treated with matched control. Subsequently, we correlated our data to gene expression using qPCR, focusing on Fgf16 and Tbx22, previously reported to be associated with cleft palate formation^3^. CCGG sequences of Fgf16 are located within the intergenic region (Chromosome X: 105725515- 105764278/ 105774940- 105797614) and include the promoter (Chromosome X: 105763200-105765801) and CCCTC binding factor (CTCF) (Chromosome X: 105762801-105763200/105,786,401-105,786,800) to indicate that regulatory regions and binding sites are differentially methylated for the Fgf16 gene. Although DNA methylation can significantly increase the rate of spontaneous C→T mutations at CpG dinucleotides and pathogenic variation [a nonsense mutation c.535C>T in exon 3 of FGF16 was identified by Jamsheer, et al.^25^ (2013)] in human FGF16 results in 4-5 metacarpal fusion (OMIM 309630), we could not find that pathogenic variation in human FGF16 results in 4-5 metacarpal fusion associated with cleft palate by a comprehensive search based on PubMed, Medline, Web of Science, and Embase databases up to December 2018. CCGG sequences of Tbx22 are located within the 5′ UTR, 3′ UTR and exons. Our qPCR results show that the methylation of these genes is inversely associated with the level of gene expression during palatal fusion.

Fgf16 encodes fibroblast growth factor 16, one of the members of the Fgf9 subfamily. The FGF, WNT, and Hedgehog signaling pathways network together in a variety of cellular processes, such as stem cell differentiation cascade, and organogenesis during embryogenesis and tissue regeneration.^26^ Recent studies have suggested that Fgf16/Fgfr2 signaling regulates palatal rugae development in the mouse's embryonic palate.^3^ Mutations in Fgf16 or Fgfr2 are associated with cleft palate, and suppression of Fgfr signaling via Fgfr kinase inhibitors causes palate defects.^27^ Potchinsky, et al.^28^ (1998) reported that several signaling pathways, for example TGFβ signaling and epidermal growth factor (EGF) signaling, are known to play significant roles in differentiating palatal tissue and converge at the MAPK cascade to regulate cellular processes^28^. Yu, et al.^29^ (2006) reported that ATRA-induced apoptosis of mouse embryonic palatal mesenchymal cells involves activation of the MAPK pathway.

Tbx22 encodes a T box-containing transcription factor that is mutated in families with X-linked cleft palate.^30^ As a transcription factor, Tbx22 is expressed specifically in palatal shelf tissues related to palatogenesis, and has been confirmed as a major genetic influence in normal palate development.^31^ Tbx22 expression disappears just prior to palatal shelf fusion and is now thought to be required for mesenchyme proliferation and shelf elevation.^32^ Sequence-specific DNA-binding transcription factors control gene expression programs in response to developmental or environmental cues.^33^ In our current study, we found that the differentially methylated CCGG sites in the Tbx22 5′ UTR, 3′ UTR and exon are hyper-methylated in the developing palate of CP mice.

Fgf16 (chromosome X: 105,764,279-105,774,939) and Tbx22 (chromosome X: 107,667,964-107,688,978) are located on the same chromosome,^34^ but Gene MANIA cannot find Fgf16 and Tbx22 to share function with it based on their interactions with it.^35^ In this study, we use three criteria to demonstrate the relationship between Fgf16 and Tbx22 epigenetics in palatogenesis following ATRA-induced cleft palate formation: 1) identification of differential DNA methylation levels from site to gene of susceptible genes, 2) identification of changes in gene expression, and 3) identification of changes in gene expression related to cleft palate *vs*. DNA methylation level. However, our current study is preliminary and much more investigation is needed to disclose the relationship between gene alterations and cleft palate formation. Our sample size is relatively small and palatal shelves were directly obtained from embryonic mouse tissues that could be mixed with other tissues. In addition, ATRA is a metabolite of vitamin A and functions to support normal pattern formation during embryogenesis, cellular differentiation and proliferation.^36^ ATRA affects global and gene-specific DNA methylation and increased expression of suppressor of variegation 3-9 homolog 2 (SUV39H2) that induces the inhibitory mark trimethylation of histone 3 lysine 9 (H3K9me3).^37^,^38^ Several studies have confirmed that ATRA induces cell cycle arrest in embryonic palatal mesenchymal (MEPM) to cause development of cleft palate.^39^ The effect of ATRA on DNA methylation level and gene expression could not be excluded, which could enhance the bias of the experimental results.

## Conclusion

In summary, our results suggest that methylation of CP-susceptible genes (Fgf16 and Tbx22) is associated with gene expression, and might be responsible for developmental failure of palatal fusion, eventually resulting in the formation of CP.
